# Active, passive, and electronic cigarette smoking is associated with asthma in adolescents

**DOI:** 10.1038/s41598-017-17958-y

**Published:** 2017-12-19

**Authors:** So Young Kim, Songyong Sim, Hyo Geun Choi

**Affiliations:** 1 Department of Otorhinolaryngology-Head & Neck Surgery, CHA Bundang Medical Center, CHA University, Seongnam, Korea; 2 Department of Statistics, Hallym University, Chuncheon, Korea; 3 Department of Otorhinolaryngology-Head & Neck Surgery, Hallym University Sacred Heart Hospital, Anyang, Korea

## Abstract

The present study investigated the associations of active, passive, and electronic cigarette (E-cigarette) smoking with asthma in Korean adolescents. We used the cross-sectional study of Korea Youth Risk Behavior Web-based Survey conducted in 2011, 2012 and 2013. Active smoking was classified into 4 groups (0 days, 1–5 days, 6–19 days, and ≥20 days a month). Passive smoking was also categorized into 4 groups (0 days, 1–2 days, 3–4 days, and ≥5 days a week). E-cigarette was defined as yes or no in the last 30 days. Age, sex, obesity, region of residence, economic level, and parental educational level were adjusted for as confounders. Smoking variables were adjusted for one another. Adjusted odd ratios (AORs) and 95% confidence intervals (CIs) were calculated using multiple logistic regression analysis with complex sampling. In total, 2.3% (4,890/216,056) of participants reported asthma in the past 12 months. Active smoking was significantly associated with asthma (AOR [95% CI] of smoking ≥20 days/month = 1.57 [1.38–1.77], P < 0.001). Passive smoking was also related with asthma (AOR [95% CI] of smoking ≥5 days/week = 1.40 [1.28–1.53], P < 0.001). E-cigarette showed positive relation with asthma, although the effects of past smoking history could not be excluded (AOR [95% CI] = 1.12 [1.01–1.26], P = 0.027).

## Introduction

An estimated 7–10% of adolescents in Korea suffer from asthma, and this prevalence is higher than that of adults (2.0%)^1,2^. Various aspects of the effects of smoking on asthma have been studied including its occurrence, aggravation, and treatment outcomes^3^. Many efforts to help individuals quit smoking have been implemented worldwide. However, active smoking continues to increase in adolescents and is currently estimated to affect approximately 14.4% of Korean male adolescents^4^. In addition, 46.5% of Korean adolescents are exposed to passive smoking in this rural male adolescent population^5^. The adverse effects of smoking are detrimental throughout the wide age range including adolescents, whose cells are actively dividing and the detoxification and repair systems remain immature^3^.

The electronic cigarette (E-cigarette) market has grown in response to the increasing concern about the health risks of smoking^6^. In addition to former or current tobacco users who transition to E-cigarette, adolescent E-cigarette smokers who have never smoked have also increased in prevalence^7^. Approximately 7.0% of Korean adolescents have been reported to be current E-cigarette smokers^8^. However, to date, very little is known about the impact of E-cigarette on asthma in adolescents. E-cigarette is related to active and passive smoking; approximately 72% of E- cigarette smokers are reported to be past smokers^9^. In addition to practicing E-cigarette instead of tobacco, the dual use of E-cigarette and tobacco smoking is also common in Korean adolescents^6^. Therefore, the confounding effects of active, passive, and E-cigarette smoking should be considered when evaluating the relationship between smoking and asthma.

The objective of the present study was to investigate the impact of active, passive, and E-cigarette smoking on asthma in Korean adolescents. This study used the doctor-diagnosed asthma to determine the prevalence of asthma. The present study extends previous studies on the associations of smoking with asthma by using a large representative adolescent population and adjusting for possible confounders including age, sex, physical activity, obesity, region of residence, economic level, parental education levels, and active, passive, and E-cigarette smoking.

## Results

In total, 2.3% (4,890/216,056) of the participants had been diagnosed with asthma in the past 12 months (Table 1). The average age of asthmatic participants was 14.7 years, which was younger than that of participants without asthma (15.0) (P < 0.001). The asthma group showed significant differences in active, passive, and E-cigarette smoking (all Ps < 0.001). Days of physical exercise (P < 0.001), sex (P < 0.001), obesity (P < 0.001), region of residence (P = 0.011), and paternal education level (P = 0.007) differed significantly between the normal and asthmatic groups, whereas economic level and maternal education level were comparable.

**Table 1:** General Characteristics of Participants.

	Normal Participants	Asthma Participants (recent 12 months)	P-value
Total Number, n (%*)	211,166 (97.7)	4,890 (2.3)	
Age, year (SD)	15.0 (1.8)	14.7 (1.8)	<0.001^†^
Physical Exercise, day/week (SD)	1.8 (2.0)	2.1 (2.1)	<0.001^†^
Sex, n (%*)			<0.001^‡^
Male	106,640 (97.3)	2,919 (2.7)	
Female	104,526 (98.1)	1,971 (1.9)	
Obesity, n (%*)			<0.001^‡^
Underweight	13,553 (97.8)	311 (2.2)	
Healthy	168,637 (97.9)	3,666 (2.1)	
Overweight	22,412 (97.0)	687 (3.0)	
Obese	6,564 (96.7)	226 (3.3)	
Region, n (%*)			0.011^‡^
Large City	96,040 (97.8)	2,128 (2.2)	
Small City	89,563 (97.7)	2,144 (2.3)	
Rural Area	25,563 (97.6)	618 (2.3)	
Economic level, n (%*)			0.494
Highest	13,578 (96.9)	438 (3.1)	
Middle High	50,428 (97.8)	1,146 (2.2)	
Middle	100,745 (98.0)	2,087 (2.0)	
Middle Low	36,475 (97.7)	863 (2.3)	
Lowest	9,940 (96.5)	356 (3.5)	
Education, Father, n (%*)			0.007^‡^
Unknown	36,518 (97.6)	907 (2.4)	
Middle School	8,835 (97.3)	243 (2.7)	
High School	93,078 (97.9)	1,975 (2.1)	
College, or over	72,735 (97.6)	1,765 (2.4)	
Education, Mother, n (%*)			0.273
Unknown	36,518 (97.6)	907 (2.4)	
Middle School	8,835 (97.3)	243 (2.7)	
High School	93,078 (97.9)	1,975 (2.1)	
College, or over	72,735 (97.6)	1,765 (2.4)	
Active Smoking, n (%*)			<0.001^‡^
0 day a month	188,708 (97.9)	4,089 (2.1)	
1–5 days a month	6,002 (96.7)	202 (3.3)	
6–19 days a month	3,455 (97.0)	108 (2.2)	
≥20 days a month	13,001 (96.4)	491 (3.6)	
Passive Smoking, n (%*)			<0.001^‡^
0 day a week	137,510 (97.9)	2,936 (2.1)	
1–2 days a week	32,477 (97.7)	759 (2.3)	
3–4 days a week	17,559 (97.6)	441 (2.5)	
≥5 days a week	23,620 (96.9)	754 (3.1)	
Electronic Cigarettes, n (%*)			<0.001^‡^
No	194,537 (97.8)	4,309 (2.2)	
Yes	16,629 (96.6)	581 (3.4)	

*Estimated prevalence adjusted recommended weighted value.

^†^Linear regression analysis with complex sampling, Significance at P < 0.05.

^‡^Chi-square test with Rao-Scott correction, Significance at P < 0.05.

The prevalence of active, passive, and E-cigarette smoking was 10.8% (23,259/216,056), 35.0% (75,600/216,056), and 8.0% (17,210/216,056), respectively. E-cigarette smokers showed significantly more active and passive smoking (all Ps < 0.001) (Table 2).

**Table 2:** Electronic cigarette smoking rates according to active and passive smoking.

	Electronic Cigarette Smoking		P-value
	No	Yes	
Active Smoking, n (%*)			<0.001^†^
0 day a month	186,212 (96.6)	6,585 (3.4)	
1–5 days a month	4,667 (75.2)	1,537 (24.8)	
6–19 days a month	2,332 (65.5)	1,231 (34.5)	
≥20 days a month	5,635 (41.8)	7,857 (58.2)	
Passive Smoking, n (%*)			<0.001^†^
0 day a week	131,920 (93.9)	8,526 (6.1)	
1–2 days a week	30,622 (92.1)	2,614 (7.9)	
3–4 days a week	15,947 (88.6)	2,053 (11.4)	
≥5 days a week	20,357 (83.5)	4,017 (16.5)	

*Estimated prevalence adjusted recommended weighted value.

^†^Chi-square test with Rao-Scott correction, Significance at P < 0.05.

Active smoking was significantly related to asthma in the past 12 months in the unadjusted model, model 1, model 2, and model 3 (all Ps < 0.001) (Table 3). A consistent positive association was noted even after adjusting for all other variables including passive smoking and E-cigarette smoking (model 3) (AOR [95% CI] for 1–5 days a month = 1.39 [1.20–1.62]; 6–19 days a month = 1.32 [1.08–1.61]; ≥20 days a month = 1.57 [1.38–1.77]). Passive smoking was also positively associated with asthma in all models (P < 0.001). The ORs for asthma were increased in accordance with the frequency of passive smoking in the unadjusted model and model 3 (AOR [95% CI] for 1–2 days a week = 1.11 [1.03–1.21]; 3–4 days a week = 1.15 [1.04–1.28]; ≥5 days a week = 1.40 [1.28–1.53]). The prevalence of asthma was significantly higher in the E-cigarette group, with an AOR of 1.13, when all other variables were considered (model 3) (95% CI = 1.01–1.26, P = 0.027).

**Table 3:** Odd ratios of active, passive and electronic cigarette smoking for asthma (recent 12 months) using multiple logistic regression analysis with complex sampling (Reference = no smoking).

Smoking	OR	95% CI	P-value
Active Smoking			
Unadjusted			<0.001*
1–5 days a month	1.55	1.34–1.80	
6–19 days a month	1.50	1.23–1.82	
≥20 days a month	1.72	1.55–1.91	
Model 1^†^		<0.001*	
1–5 days a month	1.51	1.30–1.75	
6–19 days a month	1.47	1.21–1.79	
≥20 days a month	1.84	1.65–2.05	
Model 2^‡^			<0.001*
1–5 days a month	1.48	1.27–1.71	
6–19 days a month	1.43	1.17–1.74	
≥20 days a month	1.79	1.61–1.99	
Model 3^§^			<0.001*
1–5 days a month	1.39	1.20–1.62	
6–19 days a month	1.32	1.08–1.61	
≥20 days a month	1.57	1.38–1.77	
Passive Smoking			
Unadjusted			<0.001*
1–2 days a week	1.14	1.05–1.24	
3–4 days a week	1.21	1.09–1.34	
≥5 days a week	1.52	1.39–1.65	
Model 1^†^			<0.001*
1–2 days a week	1.22	1.04–1.22	
3–4 days a week	1.21	1.09–1.34	
≥5 days a week	1.57	1.44–1.71	
Model 2^‡^			<0.001*
1–2 days a week	1.13	1.04–1.23	
3–4 days a week	1.20	1.08–1.33	
≥5 days a week	1.52	1.39–1.65	
Model 3^§^			<0.001*
1–2 days a week	1.11	1.03–1.21	
3–4 days a week	1.15	1.04–1.28	
≥5 days a week	1.40	1.28–1.53	
Electronic Cigarettes			
Unadjusted	1.54	1.41–1.69	<0.001*
Model 1^†^	1.53	1.39–1.69	<0.001*
Model 2^‡^	1.49	1.36–1.65	<0.001*
Model 3^§^	1.13	1.01–1.26	0.027*

^*^Significance at P < 0.05.

^†^Adjusted for age and sex.

^‡^Adjusted for age, physical exercise, sex, obesity, region of residence, economic level, educational level of father, and education level of mother.

^§^Adjusted for age, physical exercise, sex, obesity, region of residence, economic level, educational level of father, education level of mother, active, passive smoking, and electronic cigarette smoking.

Subgroup analysis according to sex showed that the prevalence of asthma was higher in active, passive, and E-cigarette smoking groups in both sex (Table S1). Thus, the interaction between sex and E-cigarette smoking was considered as a variable of sex*E-cigarette smoking and adjusted in model 4 (Table 4). The active and passive smoking showed positive proportional relations with asthma. The E-cigarette smoking was associated with asthma in model 4 (AOR = 1.74, 95% CI = 1.44–2.11, P < 0.001). In addition, the sex*E-cigarette smoking showed positive association with asthma (AOR = 1.33, 95% CI = 1.08–1.63, P < 0.001).

**Table 4:** Odd ratios of active, passive and electronic cigarette smoking for asthma (recent 12 months) using multiple logistic regression analysis with complex sampling (Reference = no smoking).

Smoking	OR	95% CI	P-value
Active Smoking			
Model 4^†^			<0.001*
1–5 days a month	1.39	1.19–1.61	
6–19 days a month	1.31	1.08–1.61	
≥20 days a month	1.58	1.40–1.78	
Passive Smoking			
Model 4^†^			<0.001*
1–2 days a week	1.11	1.02–1.21	
3–4 days a week	1.15	1.03–1.27	
≥5 days a week	1.39	1.28–1.52	
Electronic Cigarettes			
Model 4^†^	1.74	1.44–2.11	<0.001*
Sex*Electronic Cigarette			
Model4^†^ (reference = male)	1.33	1.08–1.63	<0.001*

^*^Significance at P < 0.05.

^†^Adjusted for age, physical exercise, obesity, region of residence, economic level, educational level of father, education level of mother, active, passive smoking, electronic cigarette smoking, sex* electronic cigarette smoking.

Both age subgroups of middle school (12–15 years old) and high school ages (15–18 years old) showed the relation between active, passive, and E-cigarette smoking and the prevalence of asthma (Table S2). The associations of active, passive, and E-cigarette smoking with lifetime asthma were also explored (Table S3). The results were consistent with those of asthma in the past 12 months, although the magnitudes of the associations (ORs) were smaller than those of past-year asthma. Active and passive smoking were positively related with the prevalence of asthma. E-cigarette also increased the occurrence of lifetime asthma in the unadjusted model, model 1, and model 2 (AOR [95% CI] = 1.12 [1.06–1.19], P < 0.001). However, lifetime asthma and E cigarette were not significantly associated after adjusting for active and passive smoking (model 3, P = 0.309).

## Discussion

In Korean adolescents, active, passive, and E-cigarette smoking in the past month were significantly more common in adolescents with asthma in the past 12 months. The associations of active and passive smoking with asthma were attenuated but still statistically significant when considering lifetime asthma.

Active smoking was positively associated with asthma in the present study. Consistent with these results, many studies have indicated adverse effects of active smoking on asthma^10–13^. The longitudinal follow up studies demonstrated the increased risk of asthma in adolescents exposed to active smoking^12,13^. Active smoking has shown positive proportional associations with asthma (ORs up to 2.0)^10^ as well as with increased late-onset asthma (>13 years) in a prospective birth cohort study^11^. Although the underlying pathophysiologic mechanism of the impact of smoking on asthma remains unclear, several plausible mechanisms have been proposed, including disturbed airway immune systems and elevated inflammatory reactions. Smoking activates neutrophils via recruitment and promotion of macrophages and immune cells^14^; furthermore, it modifies the airway microbiome primarily regarding *Haemophilus sp*., *Streptococcus sp*. and *M*. *catarrhalis* and increases its diversity, resulting in neutrophilic airway inflammation^15^. In addition, smoking affects asthma patients by generating chronic mucus hypersecretion, further exacerbation, and impaired therapeutic response^16^. Our data showed a statistically significant relationship between active smoking and lifetime asthma. This association may due to the contribution of smoking to the persistence of asthma in addition to its occurrence.

Passive smoking was also proportionally related with asthma in the present study. Several prior studies have also suggested an association between passive smoking and asthma^10,17^. Children <18 years whose mothers were active smokers had more diagnosed asthma (AOR = 1.20, 95% CI, 1.03–1.40)^17^. The plausible pathophysiologic mechanisms of the effect of passive smoking on asthma might be comparable to those of active smoking. Although both active and passive smoking exposures are composed of comparable chemicals, side strem of cigarrete smoking is largely responsible for more hazardous side-effects of tobacco smoking^18^. The effects of passive smoking on asthma have been suggested to be additive to those of active smoking^10^. Moreover, passive smoking is believed to have prolonged adverse impacts on asthma. A prospective birth cohort study reported that passive smoking during the prenatal (OR = 1.45, 95% CI = 1.15–1.83) and infant period (OR = 1.23, 95% CI = 1.01–1.51) elevated the risk of asthma in adolescence^19^. When considering chronic exposure to passive smoking from indoor active smokers, these cumulative effects of passive smoking might contribute to the statistically significant association between passive smoking and asthma identified in the present results.

This study demonstrated a positive association between E-cigarette and asthma in the past 12 months. In line with this finding, a recent study reported a significantly higher prevalence of asthma among Florida adolescents who reported E-cigarette smoking (AOR = 1.55, 95% CI = 1.17–2.05 in metropolitan residents; AOR = 2.20, 95% CI = 1.47–3.31 in rural residents)^20^. Similarly, another recent study reported a high OR of 2.74 (95% CI = 1.30–5.78) for asthma in current E-cigarette smokers who had never actively smoked among Korean adolescents^8^. However, both prior studies did not consider passive smoking exposure. This study adjusted for exposure to active or passive smoking and demonstrated a significant association between asthma and E-cigarette. However, E-cigarette smoking in the past month was not significantly associated with lifetime asthma after adjusting for active and passive smoking in the present study. Active and passive smoking were thus considered to be more influential in previous asthma history than recent E-cigarette smoking. As a high proportion of E-cigarette smokers are generally previous active smokers, the effects of previous active smoking were high in this group.

The health effects of E-cigarette are controversial. The advantages of E-cigarette compared to tobacco smoking include a reduction in several toxins present in tobacco including volatile organic compounds (1,3-butadiene, benzene, acrylonitrile) and tobacco-specific nitrosamine, although the amount of nicotine is comparable^21^. Current review data have not indicated any serious adverse health risks associated with E-cigarette^22^. However, irritation of the mouth and/or throat and the long-term effects have not been considered^22^. Additionally, the presence of propylene glycol in E-cigarette and of other toxic chemicals that originate from liquid cartridges have been suggested to be detrimental to lung function and related to asthma^23^. Further study is warranted with E-cigarette smokers who never exposed to active or passive smoking.

The strengths of this study include its large sample size, population-based design, and adjustment for numerous potential confounders. Notably, E cigarette smoking as well as active and passive smoking were considered and adjusted for reciprocally. Compared to most studies on passive smoking, which have used parental smoking patterns as a measure of passive smoking in children, the current study directly assessed the duration of exposure to passive smoking and thus more precisely measured the frequency of passive smoking. However, some limitations of the present study should be considered. The cross-sectional study design preludes the determination of causal relationships between asthma and smoking. Although several possible confounders were adjusted in this study, there are still unconcerned variables, including family history of asthma and parental smoking. The present study could not discriminate between chronic obstructive pulmonary disease and asthma. Because the survey was based on self-reported questionnaires, undiagnosed or misclassified asthma was likely. In the present study, the incidences of asthma in the past 12 months and ever were 2.3% and 9.1%, respectively. These figures were comparable to the prior Korean study which reported 3.2% and 7.7–9.1% for the asthma in the past 12 months and ever, respectively. The present study used the doctor-diagnosed asthma to prevent overestimation of asthma. However, because the questionnaire was investigated for the diagnosis of asthma from doctor, the misdiagnosed or subclinical asthma patients might be excluded in this study. Indeed, the ISSAC survey showed higher prevalence of asthma than current study. The 12-month prevalence of asthma symptom was reported about 13.2–13.7% for 13–14 years in worldwide^24,25^. The other respiratory disorders, such as chronic obstructive pulmonary disease (COPD) could be included in this study due to the indiscrimination of COPD from asthma in adolescents. However, the mean age of COPD patients were 45 years and it’s prevalence in youth was supposed to be low^26^. Regarding smoking exposures, the presence or frequency of active and E-cigarette smoking could be underestimated. The past smoking histories in E-cigarette smokers could affect to the relation between E-cigarette smoking and asthma. In addition, the intensity and duration of smoking could not be considered in the present study. However, prior reports have validated the KYRBWS questionnaire for smoking was verified using urine cotinine level of 50 ng/dl and showed 0.41–0.85 of Kappa’s coefficient and demonstrated high concordance rate^27^. Moreover, KYRBWS data have been repeatedly validated and designed to be representative of the Korean adolescent population using stratified sampling and weighted analysis.

## Conclusions

Active, passive, and E-cigarette smoking were positively associated with asthma in the past 12 months in Korean adolescents. The associations of smoking with lifetime asthma were attenuated and were significant for active and passive smoking. The effects of E-cigarette should be evaluated while considering active and passive smoking status.

## Materials and Methods

### Study Population and Data Collection

The Institutional Review Board of the Centers for Disease Control and Prevention of Korea (KCDC) approved this study (2014-06EXP-02-P-A). Written informed consent was obtained from each participant prior to the survey. As this web-based survey was performed at schools with a large number of participants, informed consent from their parents was exempted. This consent procedure was approved by the KCDC IRB. All KYRBWS data analyses were conducted in accordance with the guidelines and regulations provided by the KCDC.

This cross-sectional study used data from the Korea Youth Risk Behavior Web-based Survey (KYRBWS) and covered the nation using statistical methods based on designed sampling and adjusted weighted values. The KYRBWS obtains data from South Korean adolescents using stratified, two-stage (schools and classes) clustered sampling based on data from the Education Ministry. Sampling was weighted by statisticians, who performed post-stratification analyses and considered the non-response rates and extreme values. Data from the 2011, 2012 and 2013 KYRBWS were analyzed. Details of the sampling methods are described on the KYRBWS website^27^. The KCDC collected the data, and Korean adolescents from 7^th^ through 12^th^ grade completed the self-administered questionnaire voluntarily and anonymously. The validity and reliability of KYRBWS have been documented by other studies^28,29^.

**Figure 1:**
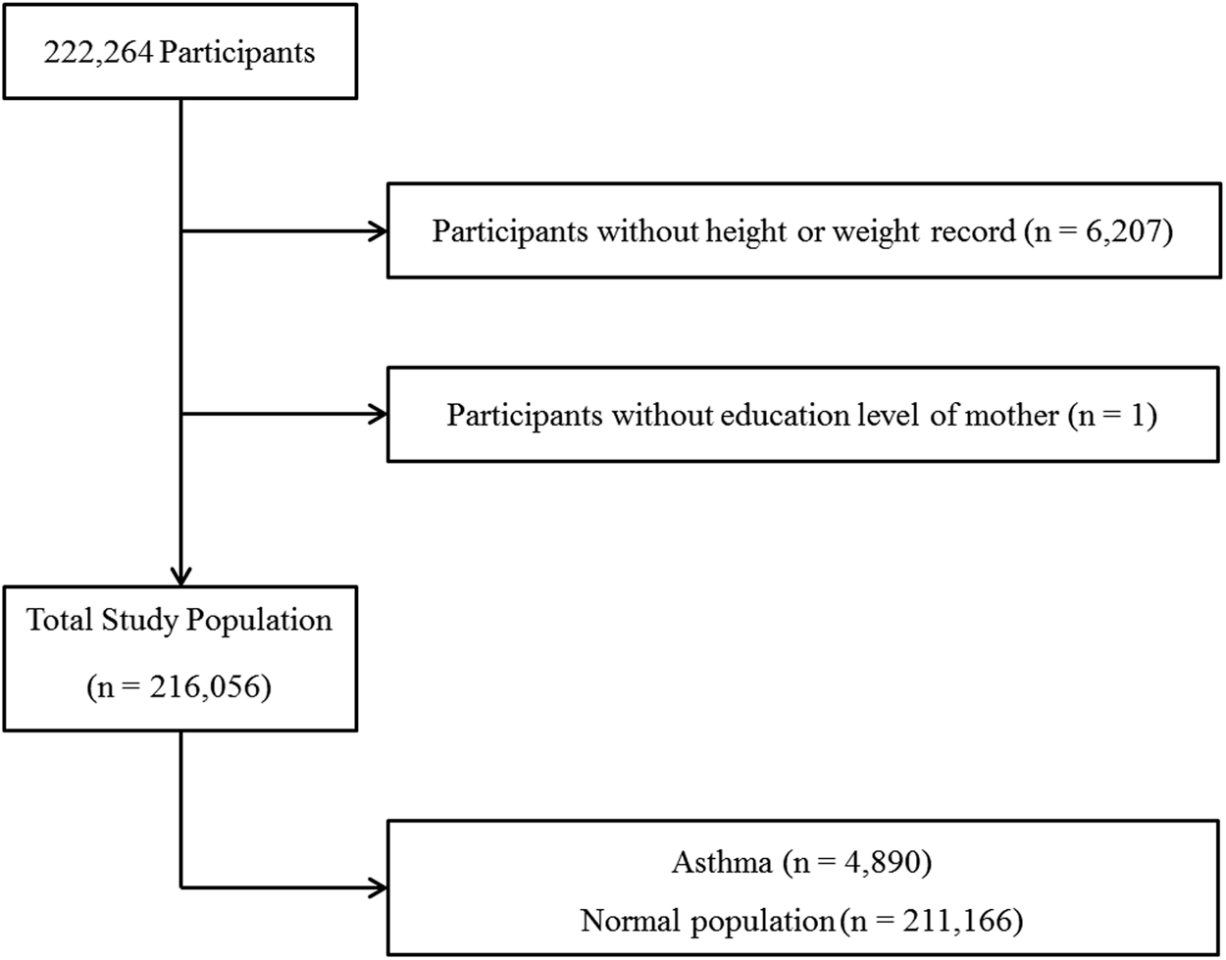
A schematic illustration of participant selection in the present study. Among a total of 222,264 participants, participants without height or weight (n = 6,207) or mothers’ educational level (n = 1) were excluded. The data for the 216,056 participants were analyzed.

Of the 222,264 total participants, the following were excluded from this study: participants without information on height or weight (6,207 participants) or mothers’ educational level (1 participant). Finally, 216,056 participants (109,559 male and 106,497 female) 12 through 18 years old were included in this study (Fig. 1).

### Survey

The understanding, reliability and validity of each question were investigated by the KCDC to verify the applicability of the surveys^27^. Days of physical activity were measured as the number of days in the past 7 days that the participants had exercised for more than 60 minutes at intensity high enough to increase their heart rate or respiration. Obesity was categorized into 4 groups according to the CDC guidelines regarding body mass index (BMI, kg/m^2^) for children and teens^30^: obese ≥95^th^ percentile; overweight ≥85^th^ percentile and <95^th^ percentile; healthy weight ≥5^th^ percentile and <85^th^ percentile; and underweight <5^th^ percentile. Region of residence was divided into 3 groups by administrative district: large city, small city, and rural area. Self-reported economic level was measured as 5 levels from highest to lowest. Parents’ education level was divided into 4 groups: graduated college or above; graduated high school; graduated middle school or lower; and unknown or no parent. Participants who did not know their parents’ educational level or who had no parents were not excluded, as their exclusion could increase the missing values of participants from a relatively lower economic level.

To measure active smoking, the participants were asked, ‘In the last 30 days, how many days did you smoke at least one cigarette?’ Active smoking was divided into 3 groups: 0 days a month, 1–9 days a month, and ≥20 days a month. To measure passive smoking, the participants were asked, ‘In the last 7 days, how many days have you been together with another person (family member or guest) who smoked in your house?’ Their responses were divided into 3 groups: 0 days a week, 1–4 days a week, and ≥5 days a week. To measure E-smoking, the participants were asked, ‘In the last 30 days, have you smoked E-cigarette?’ E-cigarette smoking was scored as yes or no.

Participants were asked about their history of the doctor-diagnosed asthma. History of asthma in the past 12 months and in their lifetime was assessed: “In the past 12 months, have you been diagnosed with asthma by a doctor?” and “Have you ever been diagnosed with asthma by a doctor?” Participants who had a history of asthma diagnosed by a medical doctor were recorded as being positive for this outcome.

### Statistical analysis

The differences in general characteristics according to asthma history (past 12 months) were calculated using linear regression analysis with complex sampling for age and days of physical activity and Chi-square tests with Rao-Scott correction for sex, obesity, region of residence, household economic level, fathers’ education level, mothers’ education level, active smoking, passive smoking, and E-cigarette smoking. The rates of E-cigarette smoking according to active and passive smoking were compared using Chi-square tests with Rao-Scott correction.

Odds ratios (ORs) and adjusted ORs (AORs) of asthma (past 12 months) based on exposure to active, passive and E-cigarette smoking were calculated using the following methods: (i) simple logistic regression analysis with complex sampling (unadjusted); (ii) multiple logistic regression analysis with complex sampling adjusted for age and sex (model 1); (iii) model 1 plus adjustments for physical exercise, obesity, region of residence, economic level, fathers’ educational level, and mothers’ education level (model 2); (iv) model 2 plus adjustments for active, passive, and E-cigarette smoking (model 3); and (v) multiple logistic regression analysis with complex sampling adjusted for age, physical exercise, obesity, region of residence, economic level, educational level of father, education level of mother, active, passive smoking, E-cigarette smoking, and sex* E-cigarette smoking (model 4).

The ORs and AORs of lifetime asthma according to active, passive and E-cigarette smoking were also calculated using the same methods.

Two-tailed analyses were conducted, and *P*-values lower than 0.05 were considered to indicate significance; 95% confidence intervals (CIs) were also calculated. The weights recommended by the KYRBWS were applied, and thus all results are presented as weighted values. To use the appropriate weighted values, missing participants were calculated as valid missing participants. The data were analyzed using SPSS ver. 21.0 (IBM, Armonk, NY, USA).

## Electronic supplementary material

Supplementary Tables
