# Supplementary material for: Active, passive, and electronic cigarette smoking is associated with asthma in adolescents
Source: Sci Rep. 2017 Dec 19;7:17789. doi: 10.1038/s41598-017-17958-y (PMC5736689; doi:10.1038/s41598-017-17958-y)
Supplement: Supplementary file 1 — Supplementary Tables [file 41598_2017_17958_MOESM1_ESM.pdf]

Active, passive, and electronic cigarette smoking is associated with asthma in adolescents

So Young Kim, MD[1], Songyong Sim, PhD[2], Hyo Geun Choi, MD[3*]

[1]Department of Otorhinolaryngology-Head & Neck Surgery, CHA Bundang Medical Center, CHA University, Seongnam, Korea

[2]Department of Statistics, Hallym University, Chuncheon, Korea

[3]Department of Otorhinolaryngology-Head & Neck Surgery, Hallym University Sacred Heart Hospital, Anyang, Korea

Corresponding. pupen@naver.com

Supplementary Table S1. Odd ratios of active, passive and electronic cigarette smoking for asthma (recent 12 months) using multiple logistic regression analysis with complex sampling (Reference = no smoking) in subgroup analyses according to sex

Smoking	OR	95% CI	P-value
Male (n = 109,559)			
Active Smoking			
Model 3†			<0.001*
1-5 days a month	1.21	1.00-1.45	
6-19 days a month	1.24	0.97-1.58	
≥ 20 days a month	1.43	1.24-1.65	
Passive Smoking			
Model 3†			<0.001*
1-2 days a week	1.13	1.01-1.26	
3-4 days a week	1.04	0.90-1.20	
≥ 5 days a week	1.30	1.16-1.47	
Electronic Cigarettes			
Model 3†	1.10	0.97-1.25	0.139
Women (n = 106,497)			
Active Smoking			
Model 3†			<0.001*
1-5 days a month	1.88	1.46-2.42	
6-19 days a month	1.55	1.10-2.19	
≥ 20 days a month	2.11	1.70-2.62	
Passive Smoking			

Model 3†			<0.001*
1-2 days a week	1.09	0.96-1.24	
3-4 days a week	1.32	1.13-1.55	
≥ 5 days a week	1.51	1.33-1.72	
Electronic Cigarettes			
Model 3†	1.42	1.15-1.76	0.001*

* Significance at P < 0.05

† Adjusted for age, physical exercise, sex, obesity, region of residence, economic level, educational level of father, education level of mother, active, passive smoking, and electronic cigarette smoking

Supplementary Table S2 Odd ratios of active, passive and electronic cigarette smoking for asthma (entire life) using multiple logistic regression analysis with complex sampling (Reference = no smoking)

Smoking	OR	95% CI	P-value
Active Smoking			
Unadjusted			<0.001*
1-5 days a month	1.10	1.01-1.20	
6-19 days a month	1.20	1.07-1.34	
≥ 20 days a month	1.17	1.10-1.24	
Model 1†			<0.001*
1-5 days a month	1.07	0.97-1.16	
6-19 days a month	1.16	1.00-1.29	
≥ 20 days a month	1.16	1.09-1.24	
Model 2‡			<0.001*
1-5 days a month	1.07	0.98-1.17	
6-19 days a month	1.16	1.03-1.29	
≥ 20 days a month	1.18	1.11-1.25	
Model 3§			0.006*
1-5 days a month	1.04	0.95-1.14	
6-19 days a month	1.12	1.00-1.25	
≥ 20 days a month	1.12	1.04-1.20	
Passive Smoking			
Unadjusted			<0.001*
1-2 days a week	1.05	1.01-1.10	

3-4 days a week	1.10	1.05-1.16	
≥ 5 days a week	1.18	1.13-1.24	
Model 1†			<0.001*
1-2 days a week	1.05	1.01-1.10	
3-4 days a week	1.11	1.05-1.17	
≥ 5 days a week	1.21	1.15-1.27	
Model 2‡			<0.001*
1-2 days a week	1.06	1.01-1.10	
3-4 days a week	1.11	1.05-1.17	
≥ 5 days a week	1.21	1.16-1.27	
Model 3§			<0.001*
1-2 days a week	1.05	1.01-1.10	
3-4 days a week	1.10	1.04-1.16	
≥ 5 days a week	1.19	1.13-1.25	
Electronic Cigarettes			
Unadjusted	1.16	1.10-1.22	<0.001*
Model 1†	1.12	1.06-1.18	<0.001*
Model 2‡	1.12	1.06-1.19	<0.001*
Model 3§	1.03	0.97-1.10	0.309

* Significance at P < 0.05

† Adjusted for age and sex

‡ Adjusted for age, physical exercise, sex, obesity, region of residence, economic level, educational level of father, and education level of mother

§ Adjusted for age, physical exercise, sex, obesity, region of residence, economic level, educational level of father, education level of mother, active, passive smoking, and electronic cigarette smoking

Supplementary Table S3 Odd ratios of active, passive and electronic cigarette smoking for asthma (recent 12 months) using multiple logistic regression analysis with complex sampling (Reference = no smoking) in subgroup analyses according to school (middle school vs. high school)

Smoking	OR	95% CI	P-value
Middle school (n = 109,373)			
Active Smoking			
Model 3†			<0.001*
1-5 days a month	1.43	1.17-1.75	
6-19 days a month	1.42	1.08-1.86	
≥ 20 days a month	1.58	1.29-1.94	
Passive Smoking			
Model 3†			<0.001*
1-2 days a week	1.06	0.96-1.18	
3-4 days a week	1.12	0.98-1.29	
≥ 5 days a week	1.43	1.28-1.60	
Electronic Cigarettes			
Model 3†	1.23	1.04-1.46	0.014*
High school (n = 106,683)			
Active Smoking			
Model 3†			<0.001*
1-5 days a month	1.37	1.09-1.71	
6-19 days a month	1.26	0.93-1.70	
≥ 20 days a month	1.64	1.40-1.92	

Passive Smoking			
Model 3†			<0.001*
1-2 days a week	1.17	1.02-1.34	
3-4 days a week	1.16	0.99-1.37	
≥ 5 days a week	1.39	1.21-1.58	
Electronic Cigarettes			
Model 3†	1.09	0.95-1.26	0.222

* Significance at P < 0.05

† Adjusted for age, physical exercise, sex, obesity, region of residence, economic level, educational level of father, education level of mother, active, passive smoking, and electronic cigarette smoking
